# Peculiarities in the designations of hepatitis B virus genes, their products, and their antigenic specificities: a potential source of misunderstandings

**DOI:** 10.1007/s11262-020-01733-9

**Published:** 2020-02-06

**Authors:** Wolfram H. Gerlich, Dieter Glebe, Anna Kramvis, Lars O. Magnius

**Affiliations:** 1 Institute for Medical Virology, Justus-Liebig University Giessen, Schubert Str. 81, 35392 Giessen, Germany; 2 Hepatitis Virus Diversity Research Unit, Department of Internal Medicine, School of Clinical Medicine, University of the Witwatersrand, 7 York Road, Parktown, Johannesburg, 2193 South Africa; 3 Ulf Lundahls Foundation, 10061 Stockholm, Sweden

**Keywords:** HBV, Subgenotypes, HBeAg, HBsAg, Subtypes, pre-C, pre-S

## Abstract

The nomenclature of the hepatitis B virus (HBV) genes and their products has developed stepwise, occasionally in an erratic way, creating many misunderstandings, especially among those who do not know the structure of HBV and its genome in detail. One of the most frequent misunderstandings, even presented in leading journals, is the designation of HBV “**e**”-antigen as *envelope* or *early* antigen. Another problem area are the so-called “**pre**” regions in the HBV genome present upstream of both the core and the surface genes of HBV, inadvertently suggesting that they may be a part of corresponding *precursor* proteins. Misnomers and misclassifications are frequent in defining the subgenotypes and serological subtypes of HBV. Even the well-established terminology for HBV surface (HBs) or HBV core (HBc) antigen deviates from the conventional virological nomenclature for viral envelopes or capsid proteins/antigens, respectively. Another matter of undesirable variability between publications is the numbering of the nucleotides and the graphical representation of genomic maps. This editorial briefly explains how the nomenclature evolved, what it really means, and suggests how it could be adapted to today’s knowledge.

## HBeAg

The motivation to write this comment was raised by an interesting article, with however, a misleading title “Serum hepatitis B virus RNA levels as an early predictor of hepatitis B *envelope* antigen seroconversion during treatment …”. The initial assumption that the authors had mixed up the HBV surface antigen (HBsAg) with the HBV “e”-antigen did not prove true: The first sentence of the abstract was: “Hepatitis B *envelope* antigen (*HBeAg*) seroconversion represents an endpoint of treatment of chronic hepatitis B virus (HBV) infections.” (*Italics* from the current authors). After asking two of the authors (who are excellent virologists and experts of HBV), both assured us that the mistake was on the side of the journal, which obviously assumed that the “**e**” represented an abbreviation for ***envelope***. This is not a rare exception. In another example, the first online version of a review article also appeared, in May 2018, with the designation hepatitis B ***envelope*** antigen for HBeAg. Fortunately, later versions contain the correct designation for hepatitis B **e**-antigen. A PubMed search for HBeAg in the last 2 years further identified 5 out of 20 articles, which contained the terms HB ***envelope*** antigen for HBeAg in the title or abstract. Another frequent misnomer is ***early*** for the **e** in HBeAg as in the first sentence of an abstract published in September 2018: “Viral biomarkers are important tools for monitoring chronic hepatitis B virus (HBV) hepatitis B ***early*** antigen (HBeAg) negative infection, … ”. Another example was found in a very recent issue of J. Virol.: “… patient HBV ***early*** antigen (HBeAg) status.”

## Meaning of the “e” in HBeAg

The somewhat *e*nigmatic name “**e**-antigen” emanates from the manner in which it was discovered. The discovery of HBV and the historical development of the hepatitis B research, during the last five decades, have been reviewed [1]. Thus, here only some details essential for the nomenclature will be mentioned and only the most historically relevant articles cited. Ironically, HB**e**Ag was discovered during studies on the hepatitis B *surface* antigen (HB**s**Ag), i.e., the major component of the HBV *envelope*. In 1971 two papers appeared in the June issue of J Infect Dis describing antigenic diversity of Australia antigen (HBsAg in today’s terminology) revealed by reactions of partial identity i.e., spur formation, in agar gel double immunodiffusion [2, 3] according to the Swedish immunologist Ouchterlony. Earlier in the same year, three antigenic specificities associated with HBV were recognized in the laboratory of one of the authors (Magnius). Two specificities, recognized by spur formation, were tentatively designated with *a* and *b*, and while another specificity that was apparently HBV-related, but showed a reaction of non-identity with the former two variants, was designated as *c*. After comparison with the antigenic determinant ***a*** and the mutually exclusive specificities ***d*** and ***y****,* published by Le Bouvier [3], the ***a*** found in Sweden corresponded to Le Bouvier’s ***a****,* and ***b*** to his ***y***, but ***c*** was new. Under the logical assumption that the new antigen was an HBV-derived antigen and continuing the sequential use of the letters of the alphabet, ***a*** to ***d***, the letter ***e*** was used for the new antigen. Explanatory words for ***e*** like *envelope* or *early* or whatever were never considered [4]. The full report on ***e***-antigen was submitted to J Immunol, but because the authors including Magnius received no response from the journal for half a year, a shorter and improved version was submitted to a Swedish Journal and in fact appeared in press before the original report [5, 6].

The same year Bancroft et al. also using agar gel double immunodiffusion recognized another pair of mutually exclusive determinants designated ***w*** and ***r*** [7]. Magnius also continued his search for additional antigenic determinants in his collection of HBsAg positive sera using agar gel double immunodiffusion and discovered the additional HBsAg determinant ***q***, which was absent in some HBV strains originating from the New World [8].

Later “**c**” was used as an obvious abbreviation for the HBV “core” antigen analogous to the “**s**” in HB**s**Ag [9]. Since in general core denotes the *interior* component of the capsid containing the nucleic acid with its associated basic proteins or polyamines, we suggest to designate “**c**” preferably as **capsid** rather than **core**, thereby keeping the abbreviations HBcAg and anti-HBc as before.

In subsequent experiments, HBeAg was determined to have a lower sedimentation constant and a higher density than HBsAg. A major part of the HBeAg in patient serum is bound to IgG and thus co-migrates with immunoglobulins [10]. This suggested that **e**-antigen behaved biophysically more like a soluble protein, and was **not** a component of a HBV-related particle, the virion, the subviral HBsAg particles or the capsid/core (HBcAg). Based on this data, Lars Magnius coined the term ***e****xtra particulate* for the **e**-antigen specificity in 1975 [10]. Indeed, it should be noted here that e-antigen remains an ***e****xtra ordinary* antigen with its unusual biogenesis (see below) and its numerous not-yet understood functions in the virus-host relationship [11].

The main point here is that the inappropriate nomenclature is not only a matter of semantics; the designation ***envelope*** is simply incorrect and highly misleading. Although the misnomer “early” antigen for HBeAg is not as grossly wrong as “envelope”, it cannot be accepted because e-antigen does **not** appear ***earlier*** than HBsAg either in HBV-infected cells or during acute HBV infections as was already noted as early as 1975, and frequently may remain positive for decades in chronic active HBV infection [12]. In fact, it is one of the viral factors contributing to the establishment for HBV persistence and its maintenance. It is true that HBeAg may disappear, in a proportion of HBV carriers earlier than other HBV markers either spontaneously or following therapy. This event is often connected with a much better immune control of the HBV infection and/or the presence of mutations [11]. This, however, is no justification for calling HBeAg “***early*** HBV antigen”.

## The gene encoding HBeAg: gene C/E

Soon it was noted that HBeAg was often present in chronic HBV patients with high viremia whereas HBV-infected patients with low or undetectable viremia often had anti-HBe antibodies [12]. This observation suggested that HBeAg may be an essential component in the viral life cycle. Yet the origin of this antigen/antibody system from host or virus and its function remained **e**nigmatic. A solution to the riddle eventually emerged step by step from the sequence of the HBV DNA. Soon after the discovery of HBeAg, a small circular partially double-stranded DNA was identified within the Dane particles (i.e., the virions) [13]. Based on this discovery, the cloning and sequencing of the entire HBV genome was underway and the groups of William Rutter [14], Pierre Tiollais [15–17], and Ken Murray [18] virtually simultaneously identified the genes encoding the HBV surface and core (or capsid) antigens (HBcAg) in 1979. Initially, HBeAg seemed to have no coding region on the HBV genome in contrast to HBsAg, HBcAg, and the HBV polymerase. (Interestingly, nobody supposed that the “X” open reading frame (ORF) might encode HBeAg). However, it was later recognized that the highly conformational epitopes of purified HBcAg extracted from the liver of HBV carriers [19] or expressed from genetically engineered *E. coli* [20] could be converted to HBeAg by denaturation, partial proteolysis, and disruption of its particulate structure in vitro. Thus, the designation “gene **C**” coined by Tiollais was inadvertently incomplete because the gene C coded not only for HBcAg but also for HBeAg and thus it might have been more appropriately called gene **C/E**. One essential difference between the core protein, present in HBV particles, and the free HBeAg in serum was identified by Takahashi et al. [21]: HBeAg contained the capsid-forming domain consisting of the 149 N-terminal amino acids of the core/capsid protein but lacked the arginine-rich nucleic acid-binding C-terminal domain of this protein. The exact N-terminus of HBeAg could not be determined in that study.

## Function of the pre-C (or *pre-E*?) region

Initially, the short 29 codon-long DNA “pre”-sequence within the ORF and upstream of gene C coding for HBcAg was not delineated in the cloned genome of HBV, with the HBsAg subtype *ayw* (Fig. 3 in Ref. [16].). Later it was included in the gene map and designated pre-C (Fig. 2a in Ref. [22].). This nomenclature was, however, somewhat unfortunate because it could suggest that the protein translated from the ORF containing the pre-C **and** C sequences would be a precursor of the mature *core* protein. In fact the opposite is correct: translation beginning with the pre-C sequence prevented the correct folding of the core protein and the assembly of core protein subunits into the capsid but favored the production of HBeAg [23]. The pre-C sequence contains a typical signal peptide directing the translated precursor protein from the cytosol to the secretory pathway, where the full-length protein P25 (with 25 kD) is first cleaved at the N-terminus in the ER to a P22 and thereafter at the C-terminus in the Golgi apparatus leading finally to secretion as the mature HBeAg P17 protein [24]. Thus, HBeAg is made from a pre-pro-protein like many other secreted proteins. This precursor contains the pre-sequence upstream of gene C, which consequently should be designated ***pre-E*** and not *pre-C*. Likewise, the mRNA encoding the HBeAg precursor protein should be designated ***pre-E* mRNA** and **not***pre-C* mRNA, because the core protein is translated from a separate mRNA, the pregenomic RNA, which lacks *pre-E*. The *pre-E* mRNA cannot serve as the pregenome because when the ribosome interacts with the *pre-E* sequence to initiate the translation of the HBeAg precursor, it opens the secondary stem-loop structure of the *pre-E* mRNA (the so-called epsilon (ε) or encapsidation signal), which is the recognition site of the viral reverse transcriptase/DNA polymerase [25]. Without this interaction, encapsidation of the pregenomic RNA and the viral polymerase cannot occur [26]. On the other hand, initiation of translation at the start codon of the core gene leaves ε functional and allows for encapsidation of the pregenomic RNA together with the polymerase, within the newly formed core protein shell [27]. It should be noted that the newly translated HBeAg pre-pro-protein P25 may not completely enter the secretory pathway [28] and the pro-protein P22 may reach the nucleus [29] where it may inhibit the expression of interferon-beta [30]. The processing of the pre-pro-protein by the ER-resident signal peptidase leaves ten amino acids of the pre-E sequence upstream of the core protein sequence [24]. This highly hydrophobic propeptide contains a Cys which interferes with the formation of intermolecular disulfide bonds stabilizing capsid formation by the HBc protein. HBe protein forms dimers like the HBc protein, but the propeptide strongly changes the shape and surface of the dimers leading to the two clearly discernible antigenicities [31].

## Alternative names for HBV antigens?

It would be a laudable attitude to avoid the steadily increasing use of unexplained abbreviations, but do we have a meaningful and correct word for “e” irrespective of the fact that “e” was not meant to be an abbreviation? HBeAg is not essential for the cellular viral life cycle, but it contributes very efficiently to the establishment of HBV persistence in perinatally infected neonates [32]. HBeAg-negative HBV variants usually cause a self-limited, often clinically unapparent infection, but occasionally can result in fatal acute hepatitis B as a result of exacerbated immune pathogenesis [33]. Thus, as the ***e****xtra particulate *version of the core protein, HBeAg may be viewed as a factor shaping the course and epidemiology of HBV infection [11].

One could argue that the term HBeAg is not needed at all because it is a second product of the gene C containing ORF. The term *core-related antigen* (**HBcrAg**) has been proposed for the epitopes present in HBeAg **and***denatured* HBcAg [34]. But this term neglects the point that HBeAg and its precursor proteins have completely opposing functions from HBcAg and the two have largely independent pathways of expression and intracellular distribution. HBeAg is not a component of the virion, while HBcAg forms the capsid and is closely linked with replication and encapsidation of HBV DNA. There are numerous clinical studies on the therapy of chronic hepatitis B, which tried to use HBcrAg instead of or in addition to HBV DNA for patient monitoring. A correlation of HBcrAg levels with the degree of liver fibrosis [35] and the development of hepatocellular carcinoma [36] was reported for HBeAg-negative patients. HBcrAg may be an acceptable substitute for HBV DNA for identification of patients requiring therapy [37], but monitoring of HBV DNA levels under antiviral therapy cannot adequately be done by HBcrAg in HBeAg-positive patients because nucleoside analogues do not suppress *transcription* of HBV DNA and the subsequent translation of HBeAg. However, HBcrAg in serum may be a better marker for HBV cccDNA in liver than HBsAg, because HBsAg can be expressed from both episomal and integrated HBV DNA, whereas HBcrAg (and HBeAg) are normally expressed only from potentially replication-competent cccDNA [38, 39].

Opinions diverge upon the functional significance of HBeAg in the biology of HBV, but its main contribution is probably the induction of immune tolerance against HBeAg and HBcAg in newborns of HBV-infected mothers [40]. This function is obviously so central to the biology of HBV-like viruses that it is conserved in most *Hepadnaviridae* species [40], although recently several HBeAg-negative species were identified in shrews [41]. Nevertheless, HBeAg is the most important marker of reduced immune reactivity during the highly replicative phase of HBV infection, while HBcAg is the strongest T and B cell antigen expressed by HBV [42]. HBeAg is in fact the basis for distinction of the various phases of chronic HBV infection together with the level of HBV replication in the current EASL guidelines [43]. The contribution of immune ***e****vasion* by HBeAg leading to the highly replicative and low inflammatory phase of HBV infection versus immune *pathogenesis,* caused by HBV replication, cannot be differentiated if HBeAg is not quantitated separately from HBcrAg. In conclusion, HBeAg cannot be replaced as a biomarker by HBcrAg because the latter generates ambiguous results in HBeAg-positive patients and cannot differentiate between high and low replicative phases.

It is remarkable that **e**-antigen received its still valid name soon after its first description in 1972 whereas it took much longer for the two essential HBV antigen systems (HBsAg and HBcAg) to be designated with generally acceptable, meaningful names in 1973/4. Originally, in 1963, HBsAg was first described as Australia antigen, in 1968 as serum hepatitis antigen (SH antigen), thereafter in several papers as hepatitis-associated antigen (HAAg) or simply HBAg, HBV coat antigen or envelope antigen. Although the term *envelope* antigen was and is scientifically correct, the possibility (or temptation) of mixing up *envelope* with the **e**-antigen makes it preferable to maintain the widely used term HBV surface antigen (HBsAg). The term *core* antigen is now generally accepted for the HBV nucleo**c**apsid although for many other viruses the term core describes the *interior* of a viral capsid. HBV capsids (with or without surface proteins) may be empty but the viable virus contains within its capsid not only the viral genome in RNA or DNA form, but also the viral polymerase and host factors like the heat shock protein 90 (Hsp90) and a host-derived protein kinase. Components, which stained positively were visible even in the very first convincing electron micrographs of HBV particles from David Dane in 1970 [44], may strictly speaking comprise the “core” or interior components of the virus albeit the nature of these components could not be identified at that time.

## The pre-S region

This designation for the 163, 173 or 174 codons (depending on the genotype of HBV [45]) upstream of the gene S encoding the HBsAg has also occasionally generated confusion. In 1979, gene S was identified encoding the then only known form of the HBsAg protein referred to as PI and its glycosylated form, PII [14–16, 18]. Soon it was realized that the ORF containing gene S was larger, and that the translational start codon on the DNA sequence encoding, the N-terminus of the major HBsAg protein was preceded by two additional conserved start codons. It was speculated that there could be a larger HBsAg precursor or another larger HBV protein [14–16, 18]. The first explicit mention of the pre-S region encoding 163 amino acids in their HBV *ayw* genome was published by Pierre Tiollais, Patrick Charnay, and Girish Vyas in 1981, who named the ORF encoding HBsAg “region S”, which was divided into “Gene S” encoding the 226 amino acid major HBsAg protein and into “Region pre-S” with unknown function (Fig. 3 in Ref. [16].). In protein biochemistry, the prefix *pre*- often means that the secreted protein is made as a precursor, from which an N-terminal signal peptide is proteolytically removed to give rise to the final form of protein. Fortunately, this somewhat suboptimal nomenclature did not create much confusion. In one article “a precursor form of HBsAg” referred to a protein, which contained additionally the peptide coded by the pre-S sequence [46] but the follow-up paper clarified that no *precursor* protein of HBsAg was identified [47].

## Functions of pre-S

**Fig. 1:**
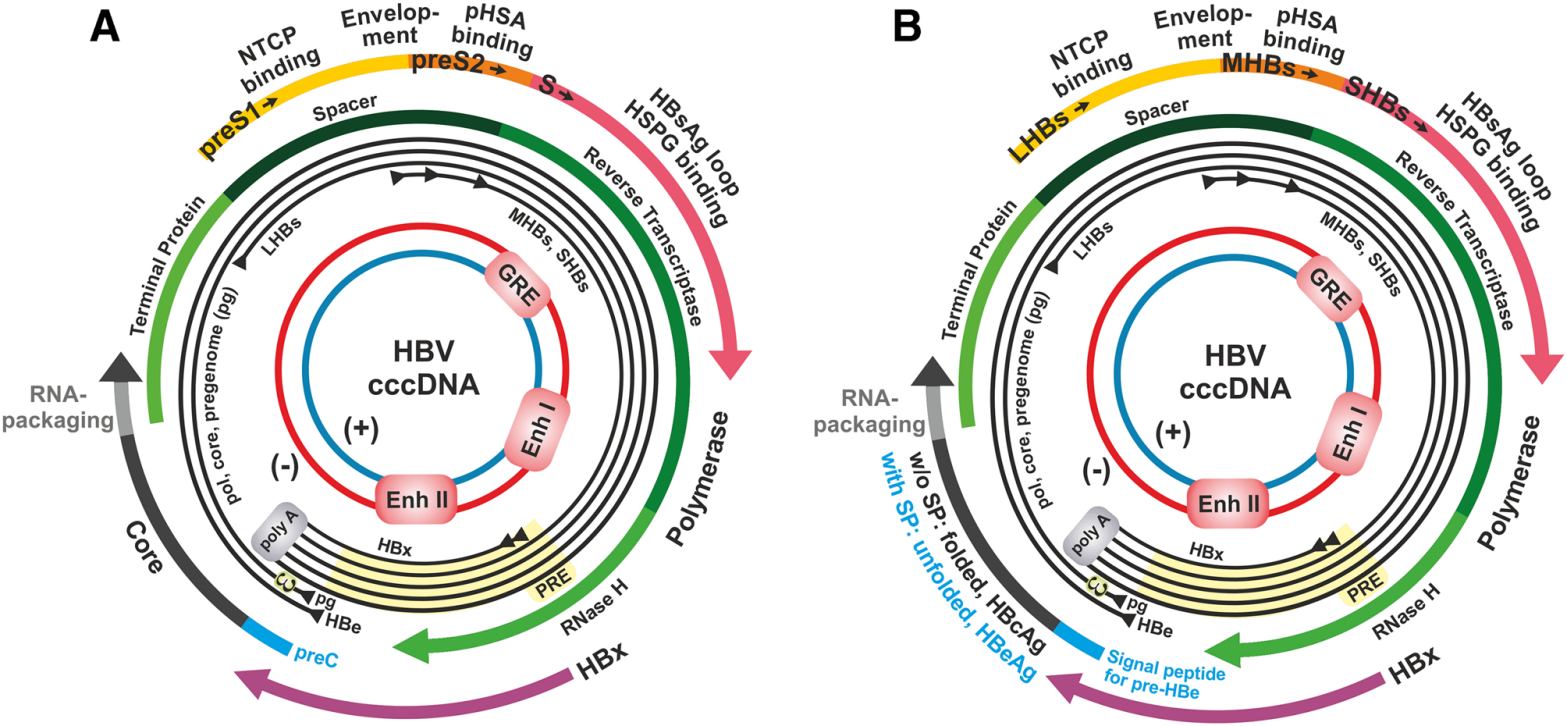
Suggested revised nomenclature for HBeAg and HBs genes, transcripts, proteins, and antigens. **a** HBV genome map with standard nomenclature taken from Ref. [83] showing the four open reading frames, their subregions, and some functions of the derived proteins on the outside. The black circles correspond to the various mRNAs, the inner closed circles show the cccDNA with enhancer I and II and the glucocorticoid response element (GRE). The EcoRI site is at the 12 h position. **b** Suggested revision of nomenclature for pre-S1, pre-S2, S domains, and pre-C sequence with corresponding mRNAs and map position of some major HBV gene functions. Note that LHBs, MHBs, and SHBs proteins have different N-termini within one ORF, but all run to a common C-terminus. The HBeAg precursor, the pre-pro-protein contains the pre-E signal peptide and the RNA packaging domain, both of which are removed during proteolytic processing. The mRNA for core protein and polymerase starts downstream of the start codon of the HBeAg pre-pro-protein and functions as the pregenomic RNA (pgRNA), which is encapsidated and reverse transcribed. The core (HBcAg) protein does not contain the pre-E signal peptide and is not glycosylated

The true functions of the pre-S region were only elucidated a number of years later. A first hint that HBsAg particles may contain pre-S encoded sequences came from the finding that HBsAg purified from the serum of HBeAg (!) positive HBV carriers contained an additional minor HBsAg protein pair [48] that was slightly larger than the well-known major protein pair P I and P II, described previously [14–16, 18]. Biochemical characterization showed that the polypeptide sequence of this minor HBsAg component started with the second conserved start codon of the S-ORF, 55 amino acids upstream of the gene encoding major HBsAg protein [49]. Interestingly, this protein pair bound an artificially generated “polymerized” human serum albumin (pHSA) [50], which was transiently considered as a potential factor for facilitating uptake of HBV by liver cells leading to the misnomer pHSA-receptor (Fig. 2 in Ref. [22].). In fact, this part of the pre-S sequence (now named pre-S2) binds a naturally modified, rare, monomeric form of HSA (mHSA) and is as a viral protein not a receptor [51]. In 1984, a more complete picture of the HBV surface was recognized when Dane particles, (i. e., complete virions), and HBsAg filaments purified from patient sera were analyzed with improved techniques and were found to contain an even larger protein pair encoded by the entire S-ORF [52]. The reasons that the two larger HBsAg protein pairs were not detected initially in HBV carriers are multiple. (i) Many HBeAg-negative HBsAg carriers contain large amounts of small subviral 20 nm particles, which consist mainly by the small (or “major”) HBs protein but lack Dane particles or the filamentous form of HBsAg. Only HBeAg-positive HBV carriers contain sufficient amounts of Dane particles or HBsAg filaments to allow the detection of the larger proteins [52, 53]. (ii) Many purification protocols lead to a proteolytic degradation of the pre-S portion of the larger HBsAg proteins. (iii) Furthermore, the HBsAg proteins do not stain well with the standard stain Coomassie Blue after sodium dodecyl sulfate (SDS) polyacrylamide gel electrophoresis, while silver staining, used in Ref. [52], readily detected the minor HBsAg proteins. In combination with the data obtained by the genome and transcription analysis, it could now be understood how the S-ORF could encode three co-carboxyterminal proteins of different size (Fig. 1) [22, 52]. Thus, the S-ORF is divided into the pre-S1, pre-S2, and S regions. The S region encodes HBsAg and the carboxyterminal ends of the large LHBs and the middle-sized MHBs proteins. LHBs is encoded by pre-S1/pre-S2/S and MHBs by pre-S2/S. It is incorrect to designate LHBs protein as pre-S1 protein because it also contains the pre-S2 and the S domains. In fact, one of the essential functions of LHBs is to interact with HBcAg particles in the cytosol and to initiate envelopment of HBV via a short domain encoded by the region located next to the junction of pre-S1 and pre-S2 [54]. Furthermore, a short domain of pre-S1 on the HBV facilitates its binding to hepatocytes and uptake leading to infection [55]. In contrast to LHBs, the expression of MHBs protein is not essential for the viral life cycle [56, 57]. Similar to HBeAg, MHBs is conserved in all mammalian HBV-like virus species (genus *Orthohepadnavirus*) and may modulate the immune response and favor persistence. Furthermore, HBeAg- or MHBs-negative HBV variants frequently evolve during chronic HBV infections [57]. Similar to, but independent of the HBeAg, assay of MHBs helps to distinguish between active and inactive forms of HBV infection which is particularly useful for monitoring of HBeAg-negative cases of chronic hepatitis B [53].

## Serological diversity of HBsAg

As will be shown, the current nomenclature of the serological HBsAg subtypes is not completely consistent. Determinant ***a*** is formed by a set of predominantly conformational disulphide-dependent epitopes, which are believed to be mainly formed by residues between position 122 and 160. Although ***a*** is by definition present in all wild-type variants of HBsAg, serological differences in addition to the ***d/y*** or ***w/r*** allele pairs soon became evident. At least four additional serological ***a*** sub-specificities—designated ***a***_***1***_, *** a***_***2***_^*1*^,*** a***_***2***_^*3*^, and ***a***_*3*_—were detected in HBsAg samples from various geographical regions [58–60]. The ***a***-sub-specificities were found together with determinants ***d*** or ***y*** and ***w***, but not in strains with ***r*** [61]. At an international workshop in 1977, the *a*-sub-specificities were therefore re-named as **w**-sub-determinants ***w1-4*** and nine HBsAg subtypes: *ayw*1, *ayw*2, *ayw*3, *ayw*4, *ayr*, *adw*2, *adw4q−, adrq*+*, adrq−* (and two very rare combinations *adyw*, *adywr*) were defined [61].

In 1987 Okamoto et al., showed that subtype determinant ***d*** corresponds to a Lys at position 122 (K122) in the small HBsAg protein (SHBs), while ***y*** has an Arg (R122); similarly ***w*** correlates with K160 and ***r*** with R160 [62]. In contrast, the molecular basis for the serological sub-specificities ***a***_***1***_, ***a***_***2***_^*1*^***, a***_***2***_^*3*^, and ***a***_*3*_ already described in 1973 by Courouce and Soulier [58–60] and in 1977 designated as ***w1*** through ***w4*** [61] could not be identified until 1992 in terms of variations in the primary S gene sequence by Norder et al. [63, 64]. The main differences between the ***a*** sub-specificities were shown to depend on substitutions of residue 127**: *w*1** and ***w*2** correlated with Pro, ***w*3** with Thr and ***w*4** with Leu or Ile [63, 64], whereas a strong interaction with the ***y*** determinant (R122) was found for **w1** and possibly also at some other positions*.* The reason for changing the nomenclature from sub-specificities of ***a*** as in Ref. [57–59] to sub-determinants of ***w*** [61] was the negative relationship between the ***a***-sub-specificities and **r**. This could finally be explained by the recognition that Ile126 in genotype C (which has mostly HBsAg subtype *adr*) has a negative effect on the reactivity of the ***a***-sub-specificities at the adjacent residue **127** and was, thus, not directly related to ***w*** [63, 64]. With this knowledge it would be logical to change the designations ***w***1 to ***w***4 into ***a1*** to ***a***4, respectively (Table 1).

**Table 1:** Previous designations of HBsAg subtypes additional to the *adw*, *ayw*, and *ayr* categories in the 1970s and a proposal for new designations based on the SHBs gene sequence

Amino acid substitutions (reference)	R^122^ P^127^	P^127^	T^127^	L/I^127^
Soulier and Couroucé [60]	*a*_*1*_	*a*_*2*_^*1*^	*a*_*2*_^*3*^	*a*_*3*_
Couroucé and Soulier [﻿61]	*w1*	*w2*	*w3*	*w4*
Gerlich et al., this study	*a1*	*a2*	*a3*	*a4*

Sub-specificity *a1* occurs only with subtype determinant *y* (i.e. **R**^**122**^) whereas *a2-4* may occur with subtype determinants *d* or *y* and *w* or *r*

Residues important for the expression of determinant ***q*** were suggested to reside at residues 177 and 178 [63, 64]. A simple algorithm for determination of the HBsAg subtype from the SHBs sequence based on reference [65] was published by Purdy et al. [66] and a bioinformatics tool for determination of serological subtypes has been made available [67].

## Genetic diversity of HBV

As briefly mentioned above, HBV can be distinguished into different strains based on sequence heterogeneity. Initially, when HBV was first cloned, the serological sub-determinants ***d/y*** and ***w/r*** of HBsAg were used to distinguish various HBV strains [14–16, 18, 22]. When complete sequences of more HBV genomes became available, they were initially classified into four genomic groups based on sequence similarity and were designated A through D by Okamoto et al. [68]. These genomic groups showed an intergroup divergence of > 8% and an intragroup divergence of < 4% based on the complete nucleotide sequence, which was thus an empirical finding rather than an arbitrary definition [68]. Using the S gene sequence alone, the intergroup divergence was > 4%, a finding that enabled the identification of the two new genomic groups E and F by Norder et al. [63]. The designation genomic group was substituted with the simpler genotype, previously defining the genetic set of an organism, now usually referred to as the genome, as opposed to the phenotype showing the physical characteristics of the organism. When a larger number of HBV genomes were subjected to pairwise comparisons, the intergroup divergence was found to be > 7.5%, which enabled the identification of genotype H [69]. Subsequently, genotypes G, I, and J have been described [70]. Divisions within the genotypes were first identified by sequencing the S region of genotype A [71] and confirmed by complete genome sequencing [72]. With between approximately 4% and 8% intergroup nucleotide divergence across the complete genome and good bootstrap support, genotypes A–D, F, and I are classified further into subgenotypes [45, 70]. When the nucleotide divergence is < 4%, then subgenotypes can be distinguished if they display distinct geographical separation (D1 vs. D2) and/or different serological subtypes (D1 (*ayw*2) vs D2 (*ayw*3), and I1 (*adw*2) vs I2 (*ayw*2), with monophyletic clustering and good bootstrap support [73].

Inconsistent use of designations is frequent in the description of HBV genetic heterogeneity. The terms “subtype” and “serotype” have been used interchangeably to describe both the HBsAg determinants and the divisions of the HBV genotypes. Thus to avoid confusion and to ensure conformity, it was earlier suggested that “serotype” or “serological subtypes” should be used synonymously to define HBsAg determinants instead of the term “subtype” [73]. “Subgenotype” replaced the terms previously used identify divisions in genotypes, including “subgroup” for genotypes A [71, 72, 74], B [74] and C [75]; “cluster” or “clade” for F [76, 77]. Roman numerals or letters previously used to number subgenotypes have been replaced with the numbers 1,2,… [45, 77]. Uniformity in the classification of subgenotypes can be achieved by applying the most updated classification criteria [70]. Recombination between HBV strains occur more often than previously recognized and this can obscure the classification criteria as can the presence of *indels* in the sequences. Therefore such sequences should be excluded from analyses. Furthermore, the range of subgenotypic divergences varies for the different genotypes. Any nomenclature will by necessity have to be modified, adapted, and optimized as new strains are characterized from different regions of the world. However, the principle of avoiding homonymia should be respected to avoid confusion in the future, i.e., any abandoned designation of a subgenotype cannot be applied to another subgenotype. In brief, following criteria should be applied for designating genotypes and subgenotypes [45, 70]:

1. A nucleotide divergence of at least 7.5% across the whole genome or 4% at the S-ORF level to separate strains into **genotypes**
2. A nucleotide divergence of between approximately 4% and 7.5%, monophyletic clustering and good bootstrap support to separate **subgenotypes**
3. Distinct geographical separation (D1 vs D2) and/or different serological subtype (D1 vs D2, and I1 vs I2), monophyletic clustering, to separate **subgenotypes**, when the nucleotide divergence is slightly lower than 4%.

The significance of the subgenotypes cannot be overestimated, because seemingly closely related subgenotypes like A1 of Africa/Asia [78] and A2 of Europe/USA differ enormously in HBeAg expression [32, 78], pathogenicity, and epidemiology [64]. Thus, description of a HBV strain remains incomplete without the accurate HBV subgenotype designation and serological subtype. Even an article published in 2018 (in a highly reputed journal) preferred to briefly characterize the HBV strain studied as *ayw* although the sequence and subgenotype (D3) was known.

Contrary to the practice in many recent publications, serological subtypes deserve designation and should be distinguished because they obviously are very immunogenic allowing detection, in the presence of the other HBsAg subtype determinants with conventional polyvalent antisera. Furthermore, they relate partially to different genotypes with characteristic geographical distribution. e. g., HBsAg *adw*2 is typical for subgenotype A2 of Europe and USA, whereas *adw*4 is typical for the phylogenetically most distant genotype F in South America. Serological subtypes *ayw*2 or *ayw*3 are typical for the Eurasian HBV genotype D whereas *ayw*4 is present in African genotype E [64, 72]. Currently, the lack of understanding of the significance of the genetic variability of HBV is illustrated by the fact that the first serendipitously selected HBV clone belonging to subgenotype A2 from USA [14] remains in most countries the basis for the HBV vaccine, although 99% of the HBV carriers globally are infected with other subgenotypes. All subgenotypes and serological subtypes contain the ‘**a**’ HBsAg determinant, which, when not extensively mutated, can be cross-neutralized by the vaccine-induced antibody. However, there are significant differences in the seroprotective potency of the vaccine against the various subgenotypes leading to frequent asymptomatic breakthroughs in countries with high HBV prevalence and subgenotypes other than A2. Breakthroughs seem to be particularly frequent with HBV strains with the HBsAg determinant ***w*4**, found in the African and South American genotypes E and F, respectively [79, 80]. These asymptomatic breakthroughs may lead to persistent occult HBV infections. Although occult HBV infections rarely cause severe disease or overt chronic infection, they are a risk factor for the development of hepatocellular carcinoma, can be transmitted by blood transfusion and can reactivate under immune suppression. Reactivated HBV variants are often heavily mutated in the HBsAg loop, thus they escape the protection provided by the hepatitis B vaccine, and can lead to frank persistence [79, 80]. This until recently, largely neglected *status quo* may endanger the goal of WHO to eliminate viral hepatitis as a public health threat by 2030.

Finally, it should be noted that alternative numbering systems of the HBV genome have been adopted over the course of time and this may create inconsistencies and confusion between earlier and later publications. In the first three papers on the sequence of the full HBV genome [14–16], it was natural to start counting clockwise at the EcoRI site and to place it on top of the circle formed by the genome as shown in Fig. 1. However, this start point is not sharply defined because the EcoRI site is somewhat variable and in many HBV strains it does not exist at all. A more consistent numbering starting with the first nucleotide of the C/E gene was somewhat later adapted by Pasek et al. [18] because this position is conserved in all viable HBV genomes. However, this numbering system was not generally accepted and the former “EcoRI” numbering is the most frequent and acceptable numbering system used nowadays. Thus we suggest that this numbering system should be preferred. The orientation of the circular HBV genome may be also presented in different ways. The first physical map of HBV DNA within virions, published from J. Summers in 1975, placed the nick and the 5′ end of the long DNA strand at the top of the circle [81]. Other authors, e.g., Seeger and Mason, also prefer placing the nick in the virion HBV DNA or the start of the core gene at the top of the circle, possibly because this is close to the “origin” of replication via reverse transcription [82]. This is in line with some other circular virus genomes where the origin of DNA replication is also placed at the top, but Seeger and Mason still started counting at the EcoRI site*.*

## Outlook

While the eminent significance of the pioneering studies on cloning and sequencing of the HBV genome remains undisputed, it is important that the designations that are used for the HBV genome and its gene products are accurate and as far as possible reflect their functional significance. Thus, it would be preferable to give the S-ORF more meaningful designations than pre-S1 or pre-S2 or S for its domains. This would avoid that newcomers in the field are misled to consider HBsAg, to be the main HBV envelope protein, whereas the proteins encoded by the pre-S to be insignificant. The P-ORF coding for the polymerase has been divided into functionally defined domains: terminal protein (tp), spacer, reverse transcriptase (rt), and RNaseH. Now that we have a better knowledge of the S domains, they can be defined in a similar fashion. Thus the S-ORF domains, beginning with the amino end, can be designated as follows (Fig. 1): bile acid transporter sodium taurocholate cotransporting polypeptide *(NTCP) binding* in LHBs, *envelopment* function in LHBs/MHBs, and *HBs antigen loop*, which binds to heparansulfate proteoglycan (HSPG) in SHBs as summarized in Ref. [83]. The lack of recognition of the HBV subgenotypes, including HBsAg subtype determinants, should be discouraged. Inconsistent terminology for the HBV genome and its genome products should be avoided as far as possible so as to prevent the misunderstanding that can arise from such misnomers. Editors and reviewers in the field must be sensitized to the inconsistencies and should ensure that the correct terminology is adhered to and used.
